# Pyrolysis of Specific Non-Recyclable Waste Materials: Energy Recovery and Detailed Product Characteristics

**DOI:** 10.3390/ma17081752

**Published:** 2024-04-11

**Authors:** Iva Janáková, Martin Čech, Šárka Grabovská, Oldřich Šigut, Pavel Sala, Agnieszka Kijo-Kleczkowska

**Affiliations:** 1 Faculty of Mining and Geology, VSB—Technical University of Ostrava, 17. Listopadu 2172/15, 708 00 Ostrava, Czech Republic; martin.cech@vsb.cz (M.Č.); grabisaris@seznam.cz (Š.G.); oldrich.sigut@vsb.cz (O.Š.); pavel.sala.st@vsb.cz (P.S.); 2 Faculty of Mechanical Engineering and Computer Science, Czestochowa University of Technology, Dabrowskiego 69, 42-201 Czestochowa, Poland; a.kijo-kleczkowska@pcz.pl

**Keywords:** pyrolysis, wood waste, plastics, automotive carpets, paper rejects, calorific value, pyrolysis products

## Abstract

This study explores the pyrolysis process applied to various non-utilized waste materials, specifically focusing on separated plastics from municipal waste, wood waste (including pallets and window frames), paper rejects, and automotive carpets. Different combinations of these waste materials were subjected to pyrolysis, a process involving high-temperature treatment (600 °C) in a nitrogen atmosphere. The resulting products, including biochar, gas, and liquid fractions, as well as the residual waste materials, underwent comprehensive analysis. The evaluation of pyrolysis products emphasizes their quality, energy content, and potential applications. Notably, the pyrolysis gas derived from the combination of separated municipal plastics and waste wood exhibited the highest calorific value at 49.45 MJ/m^3^. Additionally, Mixture 2, consisting of plastic and wood waste, demonstrated the highest calorific value for the pyrolysis condensate, reaching 30.62 MJ/kg. Moreover, Mixture 3, benefiting from biochar utilization as a sorbent, displayed the highest iodine value at 90.01 mg/g.

## 1. Introduction

For contemporary human civilization, increasing energy production and consumption of all kinds are characteristic [1]. The majority of this energy is obtained from conventional sources such as coal [2] or oil, and even though new reserves of these substances are still being discovered today, it is not sustainable to rely on them for the future [3]. The term ‘sustainable development’ is increasingly used, aiming for energy, economic, and societal sustainability while considering environmental preservation for future generations [4]. There are various ways to address this issue, such as the utilization of renewable energy sources like water, wind, geothermal energy, or solar power for heat and electricity generation [5,6].

Another problem related to the ever-increasing energy consumption is also a significant increase in the amount of waste produced [7]. Some of it can be recycled, but a large portion of the waste still ends up in landfills without further utilization, negatively impacting its surroundings. 

A sustainable economy of waste is a very important environmental aspect. In 2020, the EU adopted the circular economy action plan and aimed to ensure that the resources it uses remain in the EU economy for as long as possible, and that waste is prevented [8].

“The transition to the circular economy will be systemic, deep and transformative, in the EU and beyond. It will require an alignment and cooperation of all stakeholders at all levels—EU, national, regional and local, and international” [9].

According to [10], 4815 kg of waste were generated per EU inhabitant in 2020 (39.2% of waste were recycled and 32.2% landfilled). For comparison, waste generation in Poland was 4492 kg per capita, and in Czech Republic, 3598 kg per capita. 

In the EU, about 48% of municipal waste was recycled by material recycling and composting. For comparison, municipal waste generation in the EU was 500 kg per capita (in 2004) and 513 kg per capita (in 2022); in Poland, 256 kg per capita (in 2004) and 364 kg per capita (in 2022); and in the Czech Republic, 279 kg per capita (in 2004) and 570 kg per capita (in 2022) [11]. 

In the EU, the quantity of waste recovered—in other words, recycled, used for backfilling or incinerated with energy recovery—increased from 870 million tonnes in 2004 to 1165 million tonnes in 2020. As a result, the share of such recovery in total waste treatment rose from 45.9% in 2004 to 59.1% in 2020 [12].

In 2020, 39.9% of the total treated waste was recycled, 12.7% was backfilled, and from 6.5%, the energy was recovered. The remaining 40.9% of the waste was landfilled (32.2%), incinerated without energy recovery (0.5%) or disposed of otherwise (8.2%) [13]. 

Research by various authors shows that recycling in any form helps reduce the environmental impact, prevents waste generation, and consumes fewer natural resources. One of the options for utilizing the energy potential of these waste materials is their thermal utilization. Thanks to today’s technologies, this method is relatively environmentally friendly, but it only involves the processing and disposal of waste, or the generation of electricity and heat [14,15,16,17,18,19,20,21,22].

For example, coal mines, wanting to meet the expectations of coal quality, were forced to expand and modernize coal enrichment plants. This causes a continuous increase in waste in the form of coal sludge. The best disposal method for these wastes is their combustion or co-combustion with other fuels [19]. 

The use of polymer waste in thermal processes, by combustion methods, pyrolysis, and gasification, has energetic purposes related to the thermal utilization of waste and energy recovery, ecological purposes related to reducing gas emissions that are harmful to the environment, and economic purposes related to the partial replacement of fuels, e.g., coal [16,18,20,21,22]. It is also important to use different wastes in composites with plastics [20,21,22]. 

However, the main goal should be not only waste disposal and the utilization of thermal energy, but also the acquisition of energy-rich substances for further use. This aspect is fulfilled by pyrolytic technologies, which belong to potentially advantageous methods of material and energy processing of waste. Due to the possibility of washing the output gases and the inert reaction environment, pyrolysis also leads to a significantly lower production of sulfur and nitrogen oxides compared to the conventional low-temperature incineration of municipal waste [23,24,25,26,27,28,29,30,31,32].

In general, it can be said that any modification and recycling will have an impact on sustainability, the economy, and the environment. That is why it is so important to consider energy strategies and the circular economy, including environmental aspects in waste management and energy engineering [33,34]. The profitability of sustainable technologies should also be kept in mind because financial viability drives long-term sustainability [35,36,37,38,39]. The advanced technologies in modern manufacturing are crucial, especially because of automation in decision-making and different process optimization [40,41].

The aim of this paper is to present the research results of the pyrolysis of different waste in the context analysis of pyrolysis products, their quality and calorific value.

In the future, the authors plan to continue the thermal research of waste materials as potential fuel sources, in environmental aspects and energy efficiency.

## 2. Materials and Methods

### 2.1. Materials 

Selecting materials for the experimental part of the work was based on the anticipated suitable properties of waste or an effort to find a method for their processing and subsequent utilization. Materials with high energy potential, i.e., calorific value, are suitable waste materials for pyrolysis. The characteristic of the resulting products is also a crucial factor, especially for the energy utilization of these products. Samples separated municipal plastic, wood waste from wood, and composite materials from the automotive sector originate from ORC recycling company, the Czech Republic. The following input materials were chosen for the pyrolysis tests:

- Separated municipal plastic (plastic waste);
- Wood waste from wood recycling;
- Composite materials from the automotive sector (carpets, roof carpets);
- Paper rejects from paper recycling.

#### 2.1.1. Paper Rejects from Paper Recycling

During paper recycling, paper rejects are generated as a byproduct. We used rejects from the production of hygiene paper, which contain additives such as kaolin Al_2_(SiO_3_)_3_, talc MgSiO_3_, calcite CaCO_3_, barite BaSO_4_, and pigments such as titanium dioxide TiO_2_. The calorific value of paper rejects varies depending on the paper machine’s technological process. The calorific value of our samples was 25 MJ/kg. Paper rejects originate from the Paper Mill in Žimrovice, the Czech Republic.

#### 2.1.2. Separated Municipal Plastic

The samples consist of municipal-separated plastic obtained from collection containers after sorting PET and HDPE plastics. The sample primarily contained polyolefins and composite plastics made up of various plastic types, including Tetra Pak, which consists of a layer of paper, polyethylene, and aluminum. The composition of separated plastic is unstable and variable, but plastics are considered the most energy-efficient component of municipal waste. The calorific value of these materials ranges from 30 to 46 MJ/kg, depending on the type of plastic [42]. 

#### 2.1.3. Wood Waste from Wood Recycling

The selected sample is a mixture of wood waste. Wood waste is initially sorted by hand, and from it, other recyclable components such as glass, ferrous and non-ferrous metals, and plastics are recovered for further recycling or other use. The so-called “dead” wood waste was crushed to the desired fraction. The sample included irreparable pallets, window and door frames, wood from demolitions, and other wooden waste.

#### 2.1.4. Composite Materials from the Automotive Sector

Carpet and roof carpet samples from the automotive industry were selected. Carpets are composed of synthetic plastic fibers, such as polyethylene, polypropylene, or polyamides (nylon), and technical polyesters (polyethylene terephthalate) [43,44]. Roof carpets are made from synthetic fabrics supplemented with glass fibers [45]. Roof carpets have low recyclability. In cases where recycling is not an option, thermochemical conversion could be an alternative.

### 2.2. Sample Preparation

Due to the nature of the waste materials, it was necessary to grind the material in a single-shaft rotary shredder with hydraulic pressure, the Odes DRJ 400 (Jaroměř, Czech Republic). The material was fed onto a conveyor belt leading to the crusher itself, where it was adjusted to a size of approximately 20 × 20 mm. Grinding was performed using the IKA MF 10 basic laboratory mill (Staufen, Germany). The microfine grinder is designed for fine grinding, with the continuous universal grinder set at 4000 rpm. The desired material granularity was below 2 mm. The moisture content in the samples was determined by the gravimetric method according to ČSN P CEN/TS 15414-1 [46] using the RADWAG MA 50.R moisture analyzer (Radom, Poland), and the ash content was determined according to ČSN EN 15403 [47].

### 2.3. Analysis of Calorific Value

The ground and dried input materials, as well as the created sample mixtures, were processed into pellets using a laboratory pellet press Maasen MP150 (Möglingen, Germany), and the analysis of the calorific value was carried out using calorimetric method in a pressure vessel on the LECO AC-350 calorimeter (St. Joseph, MI, USA). The method operates on the principle of combusting the sample within a calorimeter bomb and gauging the resultant temperature rise in the water bath of the calorimeter. This process facilitates the calculation of the net calorific value (or low heating value, LHV) of fuels.

To calculate the heat of combustion, the mass of the substance burned and the temperature change in the water bath are recorded. Using the specific heat capacity of water, the heat absorbed by the water is calculated. This heat is then equated to the heat released during the combustion reaction, allowing for the determination of the heat of combustion. The determination is in accordance with EN 15357 [48].

### 2.4. Preparation of Mixtures

In total, four mixtures were prepared from the four input samples (Table 1). These mixtures were prepared in various weight ratios. Calorific value and ash content analyses were conducted on all of these mixtures. The weight proportions of the mixtures were based on the calorific value of individual waste materials. To achieve an optimal calorific value of the mixtures, we combined samples with a high calorific value, i.e., plastics, with samples of lower calorific value, i.e., wood waste, automotive waste. Waste materials were mixed in pre-selected ratios to maintain a constant volume. The pyrolysis reactor has a constant volume of 10 L.

### 2.5. Pyrolysis

**Figure 1:**
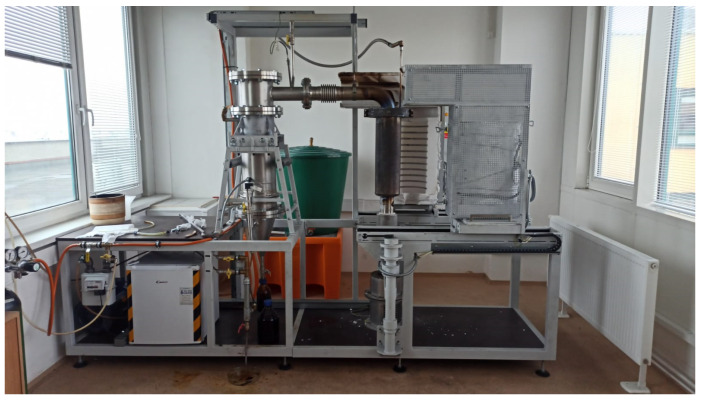
Photo of “Pyrolab 10” pyrolysis unit (VSB-TUO, Ostrava, Czech Republic).

Three experiments were conducted using the “Pyrolab 10” pyrolysis unit (VSB-TUO, Czech Republic), as shown in Figure 1. This laboratory pyrolysis unit consists of a reactor with a fixed grate that is heated by an electric spiral around the jacket. In our previous work [49], we focused on the comprehensive characterization of selected waste materials and tested various waste materials. The analyses provided very specific data regarding the modeling and optimization of the pyrolytic process, and therefore, we set the reactor temperature to 600 °C. As a result, we can expect higher-quality pyrolytic products. The collection of pyrolysis gas depended on the gas evolution during the pyrolysis process and ranged from 140 °C to 430 °C. Hot gases are directed from the reactor to a cooling device, where condensate is formed. Gas cleaning is ensured by a system of scrubbing devices.

### 2.6. Analysis of Outputs

The pyrolysis coke or solid residue from the pyrolysis test was subjected to an ash content test according to ČSN EN 15403. The sample is heated in air to a temperature of 815 ± 10 °C at a specified rate and is maintained at this temperature until a constant weight is achieved. The ash content is calculated from the weight of the residue after combustion. Further analysis of the solid residue included measuring the iodine adsorption number according to DIN 53582 [50].

Chromatographic analysis of pyrolysis gas was conducted according to the standard ČSN EN ISO 6974-6 [51] on the YL 6100 instrument (Young Lin Instrument Co, Gyeonggi-do, Republic of Korea). Using a YL 6100 gas chromatograph (Young Lin Instrument Co, Gyeonggi-do, Republic of Korea) equipped with FID and TCD detectors and a micropacked ShinCarbon column (2 m × 0.53 mm), we analyzed the abundance of CH_4_, CO_2_, H_2_, CO, and light hydrocarbons. Subsequently, based on the gas composition of each mixture, the gross calorific value was calculated.

An analysis of pyrolysis liquid (condensate) with a determination of hydrocarbons was performed on the LECO TGA 701 instrument (St. Joseph, MI, USA) according to the ASTM D7582 standard [52]. For the pyrolysis liquid analyses, 10 mL of condensate and 10 mL of dichloromethane were used as samples. The dichloromethane phase was separated using a funnel, and the remaining phase was mixed with 10 mL of diethyl ether before separating the ether phase. The solvents from both ether and dichloromethane phases were evaporated using SBHCNC/1 and SBH200D/3 sample concentrators with a Stuart block heater. Subsequently, 5 mL of C_3_H_6_O was applied to dissolve the resulting film. The solution was then analyzed using an Agilent 7890b gas chromatograph with a mass spectrometer, equipped with HP-5 and WAX columns for non-polar and polar substances, respectively. The mass spectrometer settings included a single-quad MS, a scanning range of 20–650 *m*/*z*, MS source temperature set to 230 °C, and MS quad temperature set to 150 °C.

The solid residue from the pyrolysis test underwent an ash content test according to ČSN EN 15403, and the iodine adsorption number was measured according to DIN 53582. Additionally, the solid residue was subjected to elemental analysis using the X-ray fluorescence spectrometer ED-XRF Delta Professional (Waltham, MA, USA).

## 3. Results and Discussion

### 3.1. Analysis of Input Materials and Mixtures 

Table 2 shows the amount of ash and calorific value in the examined input samples and in the prepared mixtures, with the determination of both ash content and calorific value being a crucial factor for the narrower selection of samples for pyrolysis itself.

For pyrolysis tests, Mixture 1 (a blend of paper waste and waste wood), Mixture 2 (a combination of separated municipal plastic and waste wood), and Mixture 3 (a mix of separated municipal plastic, paper waste, and waste wood) were selected, which are the samples with the lowest ash content. Mixture 2 (separated municipal plastic and waste wood) had the lowest ash content among the mixtures at 2.94%. Sample 3 (waste wood) had the overall lowest ash content (1.49%). Sample 4 (automotive carpets) exhibited the highest ash content (27.79%). The analysis of ash content indicates that Mixture 4 (24.28%) had the highest ash content.

Furthermore, all samples underwent analysis for calorific value. The highest calorific value (30.35 MJ/kg) was observed in input sample 2 (separated municipal plastic). For comparison, waste from the petrochemical industry, specifically polyolefin waste, can reach calorific values of up to 46.16 MJ/kg and a heating value of 43.00 MJ/kg [53]. The lowest calorific value was found in sample 3 at 16.96 MJ/kg (waste wood). Among the mixtures, Mixture 3 had the highest calorific value at 33.21 MJ/kg (a blend of paper waste, separated municipal plastic, and waste wood), and the lowest values were found in Mixture 1 at 18.49 MJ/kg (a blend of paper waste and waste wood).

### 3.2. Pyrolysis Tests

For the pyrolysis process itself, three sample mixtures, specifically Mixtures 1, 2, and 3, were selected. The choice of samples was based on the analysis of calorific value and the determination of ash content. The sample mixtures consist of various weight ratios of materials, which should result in different quality outputs from the process.

#### 3.2.1. Pyrolysis Test Results—Mixture 1

The first pyrolysis test was conducted with a mixture of separated municipal plastic and waste wood in a 1:1 weight ratio. The pyrolysis results are presented in Table 3.

The evolution of the process gas was very slow, and therefore, the pyrolysis gas was collected only at a reactor temperature of 430 °C when the gas evolution was sufficiently substantial, allowing for the extraction of a representative amount for subsequent analyses. The consistency of the pyrolysis oil was thin, resembling gasoline, with the rapid separation of a dark hydrophobic component that formed a centimeter-thick surface layer.

#### 3.2.2. Pyrolysis Test Results—Mixture 2

The second pyrolysis test was conducted with a mixture of paper waste and waste wood in a weight ratio of 1:3. The pyrolysis results are provided in Table 4. 

Due to the rapid release of volatile compounds from the organic material, there was a quick development of pyrolysis gas. It was collected at a reactor temperature of 140 °C. The pyrolysis oil had a thin consistency and a moderately dark color with a visibly separated hydrophobic layer (approximately 0.5 cm). The solid residue also contained unburned components. Based on visual inspection, it was likely a part of the paper waste.

#### 3.2.3. Pyrolysis Test Results—Mixture 3

The final pyrolysis test was carried out with a mixture of separated municipal plastic, paper waste, and waste wood in a weight ratio of 1:1:1. The pyrolysis results are presented in Table 5.

The evolution of pyrolysis gas was gradual, yet it had the highest volume of all pyrolyzed mixtures, amounting to 350 m^3^. Pyrolysis gas was collected at a reactor temperature of 300 °C. There was very little pyrolysis oil, and it had a thin consistency and a light color. Some “clusters” were observed in the oil, which were subjected to further analyses.

Table 6 provides an overview of the percentage yield of pyrolysis oil and gas. The gas yield was calculated based on the measured weights of pyrolysis oil, coke, and captured tars, following the law of mass conservation.

### 3.3. Analysis of Pyrolysis Output Products

#### 3.3.1. Analysis of Solid Residue (Biochar)

The solid residue was characterized by measuring the iodine number and ash content. The iodine adsorption number is used to describe the surface properties of carbonaceous sorbents, such as activated carbon. It can be said that the higher the values of the iodine adsorption number, the greater the number of double bonds present in the sorbent. This is associated with the material’s increased porosity, making physical adsorption more straightforward [54].

Table 7 records the average ash content and average iodine adsorption number of pyrolysis coke from Mixtures 1–3.

The highest ash content (33.48%) was measured in Mixture 3 (a combination of separated municipal plastic, paper waste, and waste wood), while the lowest ash content (14.44%) was found in Sample 2 (a combination of separated municipal plastic and waste wood). The average ash content values may be influenced by varying degrees of sample combustion during the pyrolysis test. The elevated ash content in Mixture 3 could be attributed to uneven combustion of the input materials. Iodine number values can be compared to values of biosorbents made from pinecone scales [55]. The highest iodine number values were measured for Sample 8 (90.01 mg/g).

Elemental analysis was further conducted on the samples. The results indicated that the most prevalent element was titanium, ranging from 2400 to 6800 mg/kg. These high values might be due to the presence of remnants of coatings with a high titanium content in the waste wood samples, serving as protection against fungi and algae. The second most prevalent element was chlorine, with values ranging from 1100 to 2400 mg/kg. Mixture 1a had the value of 1100 mg/kg, while Mixture 3 had the highest value of 2400 mg/kg. Elevated chlorine values are likely due to a higher occurrence of polyvinyl chloride in the plastic waste samples. If one intends to use the pyrolysis residue as a biosorbent, the increased chlorine values are undesirable and must be addressed with a series of procedures to improve quality and purity. However, in such a case, the economic aspect should also be considered.

#### 3.3.2. Analysis of Pyrolysis Gas

The gas underwent chromatographic analysis, allowing for the determination of the volumetric composition of individual components in the pyrolysis gas. Furthermore, the values for calorific content and heating were calculated (Table 8). From Table 8, it is evident that H_2_ had the highest representation in all samples, especially in Mixture 1. Methane, as one of the combustible gases, had a representation of approximately 20%. The lowest volumetric representation in the gas was for C_2_H_2_ and C_3_H_8_. 

The gross calorific value of pyrolysis gas from Mixtures 1–3 ranged from 35.41 MJ/m^3^ to 49.45 MJ/m^3^ (Mixture 2). For comparison with the calorific values of natural gas at 15 °C, it can be said that pyrolysis gas exhibits sufficient energy potential when compared to pipeline gas (37.72 MJ/m^3^) and Algerian natural gas (42.81 MJ/m^3^).

#### 3.3.3. Analysis of Pyrolysis Liquid

Elemental analysis was performed on the pyrolysis liquid (condensate) to determine the content of C, H, N, fixed and volatile combustibles, and moisture. All samples contained a higher amount of water, leading to phase instability. The results of the elemental analysis are succinctly summarized in Table 9. 

The sulfur content was lower than 0.6 wt%. Part of the determined moisture is attributed to the lighter components of volatile combustibles. The table also provides average calorific values for the pyrolysis liquid, with the highest net calorific value found in the condensate from Mixture 2 (30.62 MJ/kg) and the lowest in Mixture 1 (17.68 MJ/kg). Low nitrogen concentrations in the condensates are noteworthy, indicating good oil quality and thus increasing practical usability. Mixture 2 exhibits very favorable parameters, making it suitable for further applications. With the appropriate choice of distillation cut to remove light fractions, it is possible to obtain oil of maritime fuel quality or find its place in the petrochemical industry.

## 4. Conclusions

This work was conducted with the aim of analyzing and evaluating the results of the pyrolysis gasification of selected waste materials, specifically waste that no longer has further utility. These materials include paper rejects—waste generated during paper recycling, plastic waste separated from municipal plastics, waste wood from wood recycling, and carpets and roofing from the automotive industry. Proximate analyses were conducted on all these materials, leading to the selection of three mixtures designated for pyrolysis. All mixtures underwent pyrolysis at a temperature of 600 °C. The pyrolysis tests were carried out without difficulties, with only a portion of the charge remaining unburned in Sample 6 (likely paper rejects). From the product weights of the pyrolysis, the yield of pyrolysis oil and gas was determined. When comparing the yield of bio-oil with existing technologies (where biomass pyrolysis achieves 50–75% *w*/*w*), the yield from the pyrolysis tests can be considered low (14–23%). Conversely, the yield of pyrolysis gas was high (39–50%). The highest yields were observed in Mixture 2 (plastic waste and wood waste), where the yield of bio-oil was 23.34%, and pyrolysis gas was 50.67%.

Pyrolysis gas and bio-oil were mainly evaluated for their energy potential, and in many technologies, after purification, the gas is used as a process gas instead of natural gas. Bio-oil can be further refined and used as a liquid fuel. Both products had calorific values that were average to above-average, with the best values achieved for the products from the mixture of separated municipal plastic and waste wood. In terms of the energy potential of waste materials and the products produced by their pyrolysis, the selected wastes and their mixtures can be recommended for this processing method. The calorific value of these materials is on par with readily available commercial fuels such as oil and natural gas. The pyrolysis gas of the mixture of separated municipal plastics and waste wood had the highest calorific value of 49.45 MJ/m^3^. Mixture 2 (plastic waste and wood waste) had the highest calorific value of pyrolysis condensate at 30.62 MJ/kg. Due to the use of biochar as sorbent, Mixture 3 had the highest iodine value at 90.01mg/g.

Pyrolysis can be a suitable method for material and chemical recycling and for reclaiming a high proportion of raw materials from waste. Given the nature of the waste, the pyrolysis of these materials should be integrated as a preliminary process within another facility, such as a pre-processing step in an energy source utilizing pyrolysis products. From an economic perspective, we can consider incorporating a portion of the energy obtained from these waste materials as renewable energy (green energy). However, given the current state and level of boilers, investment in specialized units would be necessary, especially concerning exhaust gas cleaning. In the long term, there is a lack of transparent legislation.

## Figures and Tables

**Table 1:** Mixture preparation.

Mixture	Description	Ratio (wt)
1	Paper rejects and wood waste	1:3
2	Plastic waste and wood waste	1:1
3	Plastic waste, wood waste and paper rejects	1:1:1
4	Automotive carpets and roof carpets	1:1
5	Automotive carpets, roof carpets and plastic waste	1:1:1

**Table 2:** Ash and calorific value analysis for pyrolysis sample selection.

Sample	Designation	A^d^ [%] *	Q^daf^_s_ [MJ/kg] **
Paper rejects	1	9.35	25.09
Plastic waste	2	4.07	30.35
Wood waste	3	1.49	16.96
Automotive carpets and roof carpets	4	27.79	20.02
Paper rejects and wood waste (1:3)	Mixture 1	5.98	18.49
Plastic waste and wood waste (1:1)	Mixture 2	2.94	21.03
Plastic waste, wood waste and paper rejects (1:1:1)	Mixture 3	6.73	33.21
Automotive carpets, roof carpets and plastic waste (1:1:1)	Mixture 4	15.82	19.03

* Ash content, ** gross calorific value.

**Table 3:** Pyrolysis test results for Mixture 1.

Quantity	Unit	Volume/Mass
m_input_	g	1281
T_max_	°C	585
V_gas_	m^3^	270
V_oil_	mL	310
m_after_	g	279.5

**Table 4:** Pyrolysis test results for Mixture 2.

Quantity	Unit	Volume/Mass
m_input_	g	1251
T_max_	°C	591
V_gas_	m^3^	340
V_oil_	mL	304
m_after_	g	337

**Table 5:** Pyrolysis test results for Mixture 3.

Quantity	Unit	Volume/Mass
m_input_	g	1245
T_max_	°C	587
V_gas_	m^3^	350
V_oil_	ml	158
m_after_	g	306.5

**Table 6:** Yield of pyrolysis oil and gas.

Mixture	Yield of Pyrolytic Oil [%]	Yield of Pyrolytic Gas [%]
1	22.31	39.05
2	23.34	50.67
3	13.73	44.96

**Table 7:** Ash content and iodine adsorption number of Mixtures.

Mixture	A^d^ [%]	I [mg/g]
1	14.88	37.32
2	14.44	36.12
3	33.48	90.01

A^d^—ash content, I—iodine value.

**Table 8:** Composition of pyrolysis gas components and net calorific value.

Sample	Mixture 1	Mixture 2	Mixture 3
H_2_ [%]	31.7	18	25.3
CO [%]	19.7	15.2	15.0
CO_2_ [%]	20.1	21.1	20.4
CH_4_ [%]	20.2	22	21.7
C_2_H_2_ [%]	0.2	0.2	0.1
C_2_H_4_ [%]	4.4	8.4	7.1
C_2_H_6_ [%]	1.4	3.2	2.4
C_3_H_4_ [%]	0.03	0.03	0.02
C_3_H_6_ [%]	1.1	4.5	3.1
C_3_H_8_ [%]	0.2	0.5	0.3
H_s_ [MJ/m^3^]	35.41	49.45	45.49

H_s_—gross calorific value.

**Table 9:** Elemental analysis of sample bio-oil.

Sample	Mixture 1	Mixture 2	Mixture 3
C [wt%]	11.9	36.4	6.8
H [wt%]	9.3	9.6	9.6
N [wt%]	1.8	0.9	1.5
S [wt%]	0.6	0.4	0.4
V^daf^ [wt%] *	7.3	23.3	5.3
FC [%] **	0.9	4.2	1.2
moisture [%]	91.9	72.5	93.6
H_s_ [MJ/kg] ***	17.68	30.62	19.92

* V^daf^—volatile matter, ** FC—fixed carbon, *** H_s_—net calorific value.

## Data Availability

Data are contained within the article.
